# Real-World Mapping of Multiple Primary Carcinoma Combinations and Survival Outcomes in Shanghai, China: Retrospective Registry-Based Study

**DOI:** 10.2196/82355

**Published:** 2026-07-06

**Authors:** Jianwei Shi, Yichen Chen, Chen Chen, Xiaopan Li, Hua Jin

**Affiliations:** 1 School of Public Health, Shanghai Jiaotong University School of Medicine Shanghai China; 2 Centers for Disease Control and Prevention Shanghai China; 3 Shanghai Jing’an District Jiangning Road Community Health Service Centre Shanghai China; 4 Department of Health Management Center, Zhongshan Hospital, Shanghai Medical College of Fudan University Shanghai China; 5 Shanghai Key Laboratory of Meteorology and Health Shanghai China; 6 Department of General Practice, Yangpu Hospital, Tongji University School of Medicine Shanghai China; 7 Shanghai General Practice and Community Health Development Research Center Shanghai China

**Keywords:** multiple primary carcinoma, site, survival rate, risk, real-world maps

## Abstract

**Background:**

Multiple primary carcinomas (MPC) represent a clinically significant yet underexplored phenomenon, where patients develop more than one distinct primary malignancy. While prior studies have examined MPC within specific cancer types, comprehensive real-world patterns of primary malignancies and their subsequent primary malignancies remain limited. Moreover, the survival outcomes associated with these MPC patterns, particularly in relation to demographic and clinical characteristics, are not well characterized.

**Objective:**

This study aimed to establish the patterns and combinations of MPC across a wide range of cancers and to assess whether the mortality status of patients with MPCs varies according to their demographic characteristics and disease status.

**Methods:**

We conducted a retrospective analysis of 1560 patients with MPC in Shanghai, China, from 2002 to 2015. Data were extracted from the Shanghai Cancer Registry, with follow-up until December 2017. Cause of death was ascertained through linkage with the Shanghai Vital Registration System. The distribution of the frequency and proportion of primary carcinoma (PC) combinations were depicted, and a life table was used to calculate the 1- to 5-year survival rates. Cox regression analysis was performed to analyze the survival risk factors of the first and second PCs.

**Results:**

Among the 1560 patients (809/1560, 51.86% male and 751/1560, 48.14% female), the most frequent first PCs were colorectal, breast, and stomach cancers, while the most frequent second PCs were lung, colorectal, and stomach cancers. The most common combinations included colorectal and lung, colorectal and stomach, and colorectal and prostate. Survival rates were lowest for first PCs of skin (5 years=46.95%) and lung (5 years=41.54%) cancers, and for second PCs of pancreatic (5 year=9.13%) and liver (5 years=14.19%) cancers. A latency period of 12 months between PC diagnoses was associated with significantly higher cancer-specific mortality for both the first primary cancer (hazard ratio [HR] 3.539, 95% CI 2.822-4.438; *P*<.001) and the second primary cancer (HR 1.369, 95% CI 1.103-1.699; *P*=.004). Older age (>65 years) and advanced tumor stage (III+IV) were also significant independent risk factors for poor survival in both first PC (age: HR 2.049, 95% CI 1.689-2.485; *P*<.001; stage: HR 1.496, 95% CI 1.315-1.703; *P*<.001) and second PC (age: HR 1.575, 95% CI 1.242-1.996; *P*<.001; stage: HR 3.933, 95% CI 3.182-4.861; *P*<.001) analyses.

**Conclusions:**

This study provides a comprehensive, real-world map of MPC patterns and highlights important findings: high-risk cancer combinations and key factors associated with poorer survival, including a short interdiagnosis interval (12 months), advanced age, and advanced tumor stage. Comprehensive prevention and control strategies for MPC should be developed, and clinicians should be aware of the risks of MPC in vulnerable populations during the early diagnosis stage.

## Introduction

In recent years, studies have repeatedly corroborated the fact that patients with cancer frequently have comorbid conditions, including cardiovascular disease, obesity, metabolic disease, mental health problems, and musculoskeletal disease [1,2]. Beyond these, the development of a second primary malignant neoplasm has been increasingly recognized as a distinct challenge for survivors with cancer [3,4]. A second primary carcinoma is a new primary cancer that is histologically or clonally unrelated to the first primary carcinoma, and is associated with a poorer prognosis that can substantially compromise long-term survival [5]. There has been significant progress in the prevention, diagnosis, and treatment of various metastatic malignant tumors [6]. However, the existence of primary malignancies and subsequent primary malignancies (multiple primary carcinomas [MPCs]), which differ clinically from metastatic malignancies, is a specific comorbid condition and a current hot topic in research. According to existing studies, patients with MPC are not a recent occurrence; indeed, in 1921, a report found that 4.7% of 3000 cases of malignancy showed “multiple growth” [4,7]. In some epidemiological studies, the frequency of certain MPC was reported to be in the range of 2% to 17% [8,9]. According to the study by Buikhuisen et al [10], which was based on the nationwide Netherlands Cancer Registry from 1989 to 2018, among 2933 patients with primary carcinoma (PC), 425 consecutive primary malignancies were observed in 376 patients [10]. Clinically, MPCs are considered to be of increasing relevance and importance. However, most existing studies in Asian and Western countries analyzed the occurrence of primary cancer of a specific type of cancer, such as lung, renal cell carcinoma, and squamous cell carcinoma of the esophagus [11]. Despite the reports on the frequency of MPC for certain types of cancer, the overall pattern of MPC based on the real-world data is not well established, which may result from the relatively small survey data and sometimes includes inadequate information on diagnosis and treatment [12].

While concerns about recurrence and metastatic disease are prevalent among survivors of common cancers such as lung, colorectal, gastric, and breast cancer [13], emerging research has focused on the investigation of survival outcomes and risk factors specifically associated with second primary cancers. Using the National Cancer Institute’s Surveillance, Epidemiology, and End Results (SEER) database from 1973 to 2002, Das et al. [14] found that 216,751 patients developed primary colorectal cancer (CRC), and over a follow-up period of 1,250,687 person-years, 5,595 developed a second primary CRC, with a standardized incidence ratio of 1.36 (95% CI 1.32-1.39). Similarly, based on the SEER data, Donin et al [15] revealed that among the 1,450,837 survivors of nonpulmonary cancers, 25,472 developed second primary lung cancers at a mean follow-up of 5.7 (SD 3.6) years, and increasing age and being divorced, widowed, or separated were independent risk factors for second primary lung cancer in most primary cancer types [15]. Moreover, the period of diagnosis of the first primary CRC was also found to be an independent risk factor for a subsequent primary CRC, with a relative hazard ratio (HR) of 1.18 (95% CI 1.06-1.31) [14]. However, by reviewing the existing studies, information related to the risks of mortality for the first PC and the second PC is seldom communicated, which may reveal more opportunities in the prevention or detection of subsequent primary cancers in their early stages [16]. In China, despite some efforts to study MPC, existing research is mostly limited to high-incidence tumors such as primary lung cancer and liver cancer [17]. Comprehensive, population-based analyses delineating the spectrum of MPC across all cancer sites are scarce, with few studies leveraging long-term data from cancer registries. Similarly, there is a lack of information on the comprehensive patterns and common combinations of MPC, as well as their specific survival rates and risk factors [18].

Therefore, it is important to provide comprehensive information on the prevalence of MPC patterns, their corresponding survival rates of various cancer sites, and the risk factors for the first and second site MPC. This study aimed to (1) establish the patterns and combinations of MPC across a wide range of cancers using data from the Cancer Registry in Shanghai, China; (2) analyze the survival rates across common MPC categories in the first and second PC sites; and (3) assess whether the mortality status of patients with MPC varied according to the individuals’ characteristics and disease situation.

## Methods

### Data Source

In our study, data on the diagnosis and survival rates of MPC were collected from the Cancer Registration System of Shanghai, which collects data from 86 public health institutions in Shanghai, China. In China, the uniform standard of diagnosis and survival information of patients with cancer has been collected since the 1970s. The Cancer Registration System was gradually established and achieved complete population coverage for Shanghai residents starting in 2002 [19].

The inclusion of patient data into the registry followed a systematic procedure. According to the registry’s operational protocol, cancer cases were reported by participating institutions. Patients or their families provided informed consent for their data to be included in the population-based cancer registry and for participation in routine follow-up surveys. The survey was conducted annually to check the survival status of patients via telephone or home visit in accordance with standardized epidemiologic protocols [20]. Additionally, the survival information of patients was obtained from the coroner’s registrar and was restricted to Shanghai residents. Cause of death was ascertained through routine linkage with the Shanghai Vital Registration System, and cancer-specific mortality was defined accordingly. The quality of data was checked and evaluated based on the Guidelines for the Chinese Cancer Registration [21] and International Agency for Research on Cancer and International Association of Cancer Registries’ data quality criteria [22]. The research team had full access to the deidentified data from the Shanghai Cancer Registry for the purpose of this study.

### Data Collection

In this study, the data of patients with MPC were extracted through a systematic, multistep process according to the *ICD-10* (*International Statistical Classification of Diseases and Related Health Problems 10th Revision*) from the Cancer Registration System of Shanghai. The data included patients who were diagnosed with primary cancer (*ICD-10*: C97) in whom the second primary cancer occurred during 2002-2015.

The start year of the first primary cancer was retrospectively established as 1988, considering the data quality of the information of patients with cancer. In detail, the Shanghai Cancer Registration System began collecting complete mortality data in 1988. However, the data from 1988 to 2001 do not include full population registration data. In 2002, the Shanghai Cancer Registration System collected data from all reports of hospitals with tumor diagnostic qualifications and medical records obtained from autopsies and community follow-ups, and the data were further verified by the local Center for Disease Control and Prevention. Given that the supplementary data from 1988 to 2001 were conducive to the analysis of the first primary cancer in this article, these data were incorporated. Data cleaning also confirmed that the quality for this earlier period was acceptable, with minimal missing critical information, justifying its inclusion for a more complete analysis of the first primary cancer.

The starting year was 2002 for the second PC because the system was deemed complete at that point, and the end year was 2015. Due to the time lag in information collection and data quality control, follow-up of deaths ended on December 31, 2017. The Shanghai Cancer Registry has achieved full population coverage, and death registration is a legal requirement related to the cancellation of a resident’s household registration and property inheritance. Therefore, the survival status of patients with cancer can be accurately traced to a specific date. Consequently, in our current analysis of patients with cancer from 2002 to 2015, with the endpoint of survival outcome set as December 31, 2017, we effectively avoided underreporting cases and had no cases of survival or loss to follow-up.

Electronic data were used to compare the incidence, mortality, and survival of cancers. Detailed variables, including age, sex, cancer type, date of diagnosis, pathology, treatment, date and cause of death, tumor, node, and metastases stage (TNM stage), and registered residence, were collected. All cancers from the registration system were combined, and 26 cancer types were identified according to the *ICD-10*.

### Variables

#### MPC

MPC refers to the occurrence of two or more independent primary malignant tumors in the same individual, either simultaneously or sequentially. MPCs were identified according to established Warren and Gates criteria, which require that (1) each tumor is histologically confirmed as malignant, (2) each tumor is distinct in its pathological morphology (or, if morphologies are similar, the tumors are anatomically separate), and (3) each tumor is neither a metastasis nor a recurrence of any prior tumor. According to the temporal sequence, MPCs were categorized based on the interval between diagnoses [23].

In this study, we observed two types of MPCs: patients with 2 PCs (first PC [site A] + second PC [site B]), and those with 3 PCs (first PC [site A] + second PC [site B] + third PC [site C]). Due to the low number of PCs (3), we focused on sites A and B when analyzing the pattern, combination, and survival rate using Cox regression.

#### Demographic Variables of Patients With MPC

We collected data on the following demographics: sex (male and female), age (65 years and 65 years, indicating the older adult group and non–older adult group, respectively), TNM (tumor, node, metastases) stage of cancer (in stage of III and IV, I, and II), diagnosis time (2002-2008, 2009-2015; 2008 was chosen because it was the median of this period), the level of the therapeutic hospital where the treatment was received (secondary hospital vs tertiary hospital in China, all patients with cancer are treated at secondary or tertiary hospitals when they were first diagnosed, since the large hospitals have sufficient expertise in diagnosing tumors), latency between the first and second PC (12 months or 12 months), and number of PCs (2 or 3).

### Statistical Analysis

The distribution of the frequency and proportion of first and second PCs was depicted using Adobe Illustrator CS5. A life table method was used to calculate the 1- to 5-year survival rates. Kaplan-Meier analysis was used to estimate the patients’ survival status, and Cox regression was performed to identify the risk factors associated with survival, for both first and second PCs. These conventional methods were selected as the primary analytical tools given the descriptive nature of this real-world mapping study. Competing risk regression was not applied, as our primary aim was to describe MPC combination patterns and identify associated survival risk factors in this real-world cohort, rather than to estimate the cumulative incidence function. Cases with missing data for the TNM stage variable were excluded from the Cox regression analyses.

All statistical analyses were 2-sided and performed using the Statistical Package for the Social Sciences software (version 20.0; SPSS, Inc). Statistical significance was set at *P*<.05 (2-sided).

### Reporting Guidelines

Results are presented in accordance with the TRIPOD (Transparent Reporting of a multivariable Prediction model for individual Prognosis or Diagnosis) guidelines. STROBE (Strengthening the Reporting of Observational Studies in Epidemiology) and RECORD (Reporting of studies Conducted using Observational Routinely collected health Data) guidelines for observational studies and studies using routinely collected health data were also considered. The study was conducted in accordance with relevant institutional guidelines. The completed RECORD checklist is provided in Multimedia Appendix 1.

### Ethical Considerations

This retrospective registry-based study used existing, deidentified data from the Shanghai Cancer Registry. The original data collection for the registry was conducted after the approval of the Institutional Review Board of Shanghai Municipal Center for Disease Control and Prevention, as part of the legally mandated population-based cancer surveillance system authorized by Shanghai public health authorities. For the purpose of this specific research analysis, the study protocol conformed to the ethical guidelines of the 1975 Declaration of Helsinki and was approved by the Ethics Committee of Fudan University, Shanghai, China (IRB00002408 and FWA00002399). The need for additional informed consent for this retrospective analysis was waived by the Ethics Committee, as the study involved the analysis of anonymized data collected for public health purposes. All data were handled confidentially, and no compensation was provided.

## Results

### Overview

**Figure 1:**
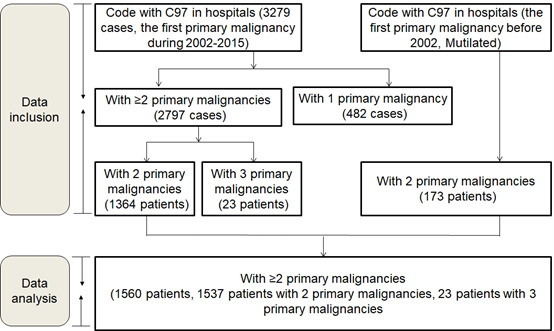
Flowchart of patient selection and data sources for the retrospective registry-based study of multiple primary carcinoma in Shanghai, China.

In this study, data from 1387 patients (2797 cancer cases; cases describing the site of cancer) included the type of the first PC. In addition, 1364 patients (2728 cases) had 2 PCs, and 23 patients (69 cases) had 3 PCs. In total, 173 patients (all of whom had 2 PCs) had their first PC before 2002. Our dataset included a total of 1560 patients with ≥ 2 PCs ([1364 with 2 PCs from 2002 to 2015] + 23 [with 3 PCs from 2002 to 2015] + 173 [with 2 PCs, with the first occurring before 2002]) for the analysis of MPC patterns and survival rates. The median follow-up time for the first and second PC groups was 6.86 years (IQR 4.42-12.18) years and 2.23 (IQR 1.46-5.39) years, respectively (Figure 1).

As shown in Table 1, among the 1560 patients with MPC (1537 with 2 PCs and 23 with 3 PCs), the distribution of male (809/1560, 51.86%) and female (751/1560, 48.14%) patients were similar. The proportion of patients >65 years of age was greater in the 2 PCs group (≤65 years, 590/1560, 38.39%; >65 years, 947/1560, 61.61%; *P*<.001). There was a higher number of patients with I + II with 2 PCs (652/1537, 42.42%) and 3 PCs (11/23, 47.83%) compared to those with III + IV PCs (2 PCs, 249/1537, 16.20%, *P*=.004; 3 PCs, 4/23, 17.39%, *P*=.88). In both groups with 2 and 3 PCs, there were higher proportions of patients who were diagnosed in 2009-2015 (2 PCs, 944/1537, 61.42%, *P*=.40; 3 PCs, 15/23, 65.22%, *P*=.02) and in secondary hospitals (2 PCs, 1067/1537, 69.42%, *P*=.44; 3 PCs, 15/23, 65.22%, *P*=.88). In addition, our results showed that 74.50% (1145/1537) of patients had a 12-month interval between the first and second PC diagnosis in the 2 PCs group and 78.26% (18/23) of patients in the 3 PCs group.

**Table 1:** Baseline demographic and clinical characteristics of 1560 patients with multiple primary carcinoma in Shanghai, China (2002-2015).

Variables		With two primary carcinomas (n=1537)						With three primary carcinomas (n=23)										Total (n=1560)	
		Male	Female	Total	Chi-square (*df*)	*P* value	Male		Female	Total	Chi-square (*df*)		*P* value			Total			
**Age (years), n (%)**					64.19 (1)	<.001						0.17 (1)			.28				
	≤65 years	230 (28.82)	360 (48.71)	590 (38.39)			3 (27.27)		7 (58.33)	10 (43.48)						600 (38.46)			
	>65 years	568 (71.18)	379 (51.29)	947 (61.61)			8 (72.73)		5 (41.67)	13 (56.52)						960 (61.54)			
**TNM^a^** **stage, n (%)**					8.18 (2)	.004						0.02 (2)		.88					
	III+IV	143 (17.92)	106 (14.34)	249 (16.20)			2 (18.18)		2 (16.67)	4 (17.39)						253 (16.22)			
	I+II	305 (38.22)	347 (46.96)	652 (42.42)			5 (45.45)		6 (50)	11 (47.83)						663 (42.50)			
	Unknown	350 (43.86)	286 (38.70)	636 (41.38)			4 (36.36)		4 (33.33)	8 (34.78)						644 (41.28)			
**Diagnosis time (** **years** **)** **, n (%)**					0.73 (1)	.40						0.02 (1)			.88				
	2009-2015	482 (60.40)	462 (62.52)	944 (61.42)			7 (63.64)		8 (66.67)	15 (65.22)						959 (61.47)			
	2002-2008	316 (39.60)	277 (37.48)	593 (38.58)			4 (36.36)		4 (33.33)	8 (34.78)						601 (38.53)			
**Therapeutic hospital level** **, n (%)**					0.61 (1)	.44						0.02 (1)			.88				
	Secondary	561 (70.30)	506 (68.47)	1067 (69.42)			7 (63.64)		8 (66.67)	15 (65.22)						1082 (69.36)			
	Tertiary	237 (29.70)	233 (31.53)	470 (30.58)			4 (36.36)		4 (33.33)	8 (34.78)						478 (30.64)			
**Latency** **, n (%)**					2.89 (1)	.09						0.38 (1)			.54				
	≤12 months	580 (72.68)	565 (76.45)	1145 (74.50)			8 (72.73)		10 (83.33)	18 (78.26)						1163 (74.55)			
	>12 months	218 (27.32)	174 (23.55)	392 (25.50)			3 (27.27)		2 (16.67)	5 (21.74)						397 (25.45)			

^a^TNM: tumor, node, and metastases stage.

### MPC Combination Patterns in the First and Second PC Sites

**Figure 2:**
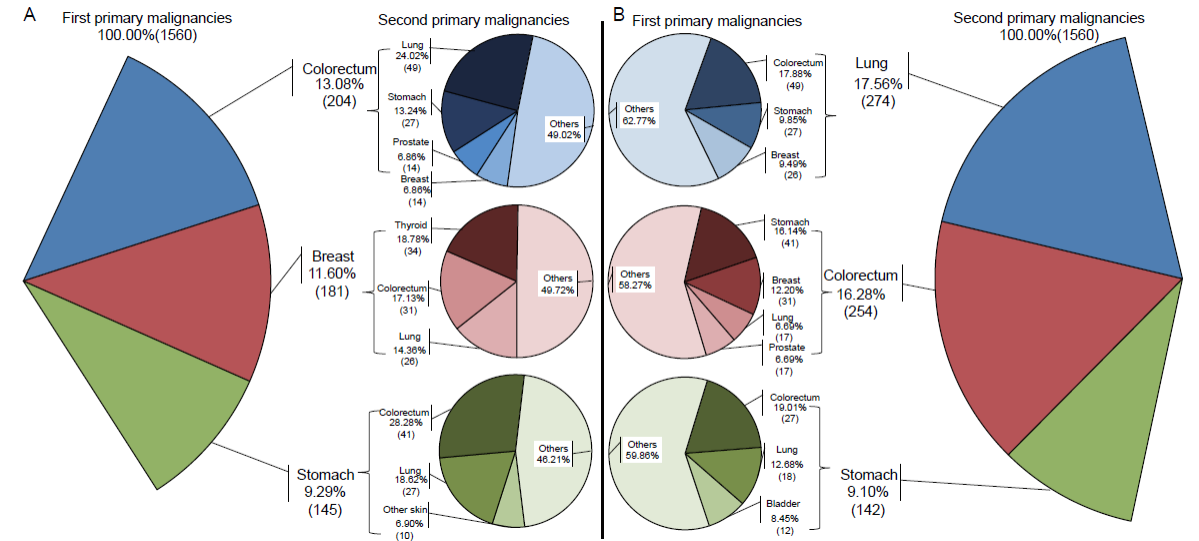
Distribution of first and second primary carcinoma sites among 1560 patients with multiple primary carcinoma in Shanghai, China (2002-2015).

Figure 2 indicates the specific cancer distribution among the 1560 patients with MPC. The results show that the top three first PCs were accumulated in the colon (204/1560, 13.08%), breast (181/1560, 11.60%), and stomach (145/1560, 9.29%). With CRC as the first PC, the second PCs included the lung (49/1560, 24.02%), stomach (27/1560, 13.24%), prostate (14/1560, 6.86%), and breast (14/1560, 6.86%). For patients with breast cancer as the first PC, thyroid (34/1560, 18.78%), colorectal (31/1560, 17.13%), and lung (26/1560, 14.36%) cancers accounted for the highest proportions of second PCs. Stomach cancers as the first PC most commonly occurred with colorectal (41/1560, 28.28%), lung (27/1560, 18.62%), and skin (10/1560, 6.90%) cancer as second PCs. The patterns of all cancers are shown in Multimedia Appendix 2.

The top three secondary PCs were lung cancer (274/1560, 17.56%), colorectal cancer (254/1560, 16.28%), and stomach cancer (142/1560, 9.10%), which collectively accounted for 42.94% (670/1560) of all secondary PCs. The most frequently observed combinations with lung cancer as the secondary PC were colorectal and lung cancer (49/274, 17.88%), stomach and lung cancer (27/274, 9.85%), and breast and lung cancer (26/274, 9.49%). Overall, stomach and colorectal cancer (41/254, 16.14%) and breast and colorectal cancer (31/254, 12.20%) were the most frequently observed MPC combinations. With stomach cancers as the secondary PC, colorectal and stomach (27/142, 19.01%), lung and stomach (18/142, 12.68%), and bladder and stomach (12/142, 8.45%) cancers accounted for the highest percentage. The complete combination patterns of all cancers are shown in Multimedia Appendix 3.

**Figure 3:**
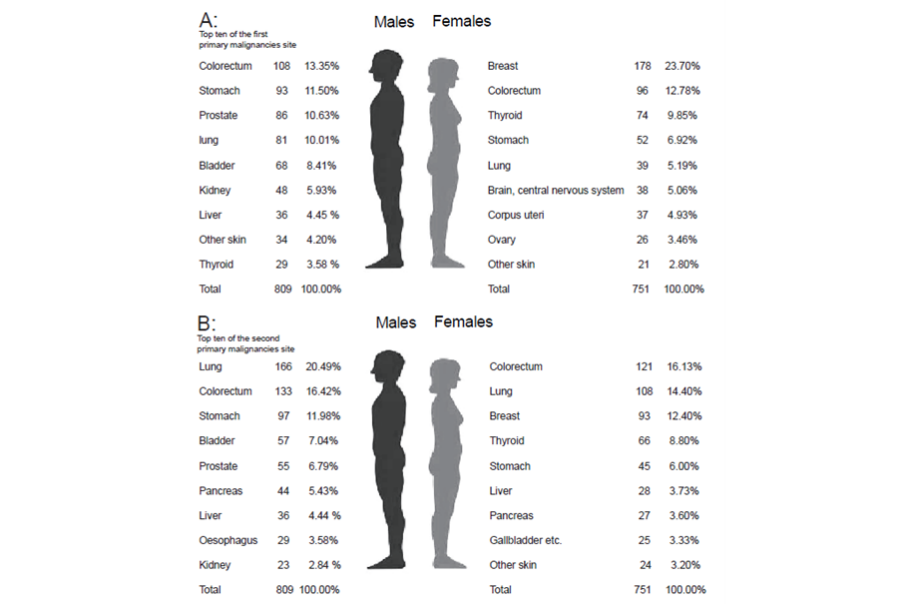
Sex-specific distribution of first and second primary carcinoma sites among 1560 patients with MPC in Shanghai, China (2002-2015). A indicates the first PC, and B indicates the second PC.

As depicted in Figure 3, the colon, stomach, and lung were the most frequently observed first PC sites for both male and female patients. Cancers of the reproductive system were also frequently diagnosed, with prostate cancer ranked the third most frequently observed (86/809, 10.63%) in male patients, and breast cancer ranked first (178/751, 23.70%) in female patients. Moreover, thyroid (ranked third), brain and central nervous system (ranked sixth), corpus uteri (ranked seventh), and ovary (ranked eighth) cancers were more common in female patients, while bladder (ranked fifth), kidney (ranked sixth), and liver cancers (ranked seventh) were more common in male patients. Lung cancer was the most frequently diagnosed secondary PC in men (166/809, 20.49%) and the second most frequent in women (108/751, 14.40%). Male patients also demonstrated a higher rate of esophageal cancer (ranked eighth) as a second PC than as a first PC. In women, liver (ranked sixth), pancreatic (ranked seventh), and gallbladder (ranked eighth) cancers were more commonly observed as second PCs than first PCs.

### Survival Rates According to the Type of Cancer in the First and Second PC Sites

The survival rates for the first and second PCs are displayed in Table 2. Focusing on the first PC site, patients with breast cancer had the highest 1-year survival rate (96.69%). Patients with thyroid cancer as the first PC had the longest 2-year (93.12%), 3-year (90.01%), 4-year (86.76%), and 5-year (83.09%) survival rates. Although ranked 10th most frequently observed, patients with brain and central nervous system cancer as the first PC had relatively high 1- and 2-year survival rates. Patients with kidney cancer as the first PC had high 3-, 4-, and 5-year survival rates. It is worth noting that skin and lung cancers were associated with the lowest survival rates in patients.

Similarly, patients with thyroid cancer as the second PC had the highest survival rates (1-year, 95.04%; 2-year, 95.04%; 3-year, 90.77%; 4-year, 84.61%; 5-year, 84.61%), followed by breast cancer. Comparatively, patients with pancreatic cancer as the second PC had the lowest survival rates (1-year, 16.90%; 2-year, 14.08%; 3-year, 14.08%; 4-year, 11.74%; 5-year, 9.13%), which were similar to those of liver cancer as the second PC (1-year, 32.81%; 2-year, 26.08%; 3-year, 17.74%; 4-year, 17.74%; 5-year, 14.19%).

**Table 2:** One- to five-year survival rates for first and second primary carcinoma sites among patients with multiple primary carcinomas in Shanghai, China (2002-2015).

	First primary carcinoma site		Survival rates (%)						Second primary carcinoma site			Survival rates (%)				
Rank	Sites	N	1-year	2-year	3-year	4-year	5-year	Sites		N	1-year		2-year	3-year	4-year	5-year
1	Colorectum	204	88.24	85.28	81.26	73.40	66.20	Lung		274	44.14		35.77	31.05	27.78	25.99
2	Breast	181	96.69	92.81	90.01	85.99	81.72	Colorectum		254	66.39		55.04	47.21	40.36	37.37
3	Stomach	145	84.04	80.47	76.04	71.50	69.17	Stomach		142	37.67		33.75	29.12	25.76	20.61
4	Lung	120	69.70	61.91	57.36	49.65	41.54	Breast		98	85.49		84.35	78.95	75.59	68.62
5	Thyroid	103	95.11	93.12	90.01	86.76	83.09	Thyroid		81	95.04		95.04	90.77	84.61	84.61
6	Prostate	86	85.99	77.69	67.98	62.89	57.60	Bladder		72	62.43		55.86	51.64	51.64	44.52
7	Bladder	80	83.75	76.25	76.25	69.73	67.02	Pancreas		71	16.90		14.08	14.08	11.74	9.13
8	Kidney	65	92.31	89.23	87.64	82.43	80.56	Liver		64	32.81		26.08	17.74	17.74	14.19
9	Other skin	55	76.36	67.27	60.00	54.46	46.95	Prostate		55	67.13		58.74	53.27	37.49	37.49
10	Brain, CNS^a^	51	94.12	90.11	86.02	81.92	75.78	Other skin		43	74.10		63.12	42.08	37.40	32.06
—^b^	Total	1560	85.28	80.35	76.41	70.85	65.62	Total		1560	56.18		49.03	43.06	39.30	36.19
—	Patients with 2 PCs^c^	1537	85.19	80.18	76.25	70.96	65.78	Patients with 2 PCs		1537	55.84		48.64	42.73	39.38	36.67
—	Patients with 3 PCs	23	91.30	91.30	86.96	65.22	56.52	Patients with 3 PCs		23	73.91		69.57	60.59	41.20	24.72

^a^CNS: central nervous system.

^d^Not applicable.

^c^PC: primary carcinoma.

### Possible Risk Factors for Survival Status in the First and Second PC Sites

The Kaplan-Meier analyses show that there were significant differences in overall and cancer-specific survival rates with regard to the first PC between sex, age, TNM, and diagnosis time and latency (*P*<.001; Figure S1A in Multimedia Appendix 4). The survival disparity between latency >12 months and < 12 months was the most sizeable. When assessing the second PC (Figure S1B in Multimedia Appendix 4), there was no significant survival difference with diagnosis time as the variable (*P*>.05), but the survival rates differed significantly based on the level of hospital where treatment was sought (*P*<.001).

Table 3 demonstrates the results of Cox regression analysis, showing that for the first PC site, there was a significantly higher HR of cancer-specific mortality in the >65-year-old group (HR 2.049, 95% CI 1.689-2.485; *P*<.001) in the patients with stage III+IV (HR 1.496, 95% CI 1.315-1.703; *P*<.001), with diagnosis times between 2009 and 2015 (HR 2.377, 95% CI 1.874-3.015; *P*<.001). Similar results were observed when assessing all-cause mortality. For the second PC, patients with an age >65 years (HR 1.575, 95% CI 1.242-1.996; *P*<.001) and at stage III+IV (HR 3.933, 95% CI 3.182-4.861; *P*<.001) also showed a higher risk of cancer-specific mortality, as well as all-cause mortality. There were also fewer male patients with higher HR cancer-specific mortality (HR 0.691, 95% CI 0.568-0.840; *P*<.001). Notably, patients with an interdiagnosis interval of ≤12 months had a significantly higher risk of both cancer-specific and all-cause mortality from the first primary cancer (cancer-specific: HR 3.539, 95% CI 2.822-4.438; all-cause: HR 3.533, 95% CI 2.824-4.420) and from the second primary cancer (cancer-specific: HR 1.369, 95% CI 1.103-1.699; all-cause: HR 1.339, 95% CI 1.081-1.659), compared to those with an interval of >12 months. Regarding the number of primary cancers, there was no significant difference between the mortality of patients with primary malignancies in the first site and second site (*P*>.05).

**Table 3:** Cox regression analysis of risk factors for cancer-specific and all-cause mortality in first and second primary carcinoma sites among patients with multiple primary carcinoma^a^.

Variables			Cancer-specific mortality					All-cause mortality			
			HR (95% CI)		*P* value		HR (95% CI)				*P* value
**In the first primary** **carcinoma** **site (** **n=916)**											
	Sex (male vs female)	0.842 (0.705-1.005)		.06		0.849 (0.713-1.011)				.07	
	Age (>65 vs ≤65 years)	2.049 (1.689-2.485)		<.001		2.092 (1.728-2.532)				<.001	
	TNM^b^ stage (III + IV vs I + II)	1.496 (1.315-1.703)		<.001		1.497 (1.318-1.700)				<.001	
	Diagnosis time (2009-2015 vs 2002-2008)	2.377 (1.874-3.015)		<.001		2.302 (1.820-2.913)				<.001	
	Therapeutic hospital level (secondary vs tertiary)	1.143 (0.937-1.395)		.19		1.149 (0.945-1.399)				.16	
	Latency (≤12 months vs >12 months)	3.539 (2.822-4.438)		<.001		3.533 (2.824-4.420)				<.001	
	Number of PCs^c^ (with 3 PCs vs with 2 PCs)	1.253 (0.667-2.353)		.48		1.212 (0.645-2.275)				.55	
**In the second primary** **carcinoma** **site (n** **=** **844)**											
	Sex (male vs female)	0.691 (0.568-0.840)		<.001		0.705 (0.581-0.855)			<.001		
	Age (>65 vs ≤65 years)	1.575 (1.242-1.996)		<.001		1.562 (1.236-1.973)			<.001		
	TNM stage (III + IV vs I + II)	3.933 (3.182-4.861)		<.001		3.837 (3.114-4.729)			<.001		
	Diagnosis time (2009-2015 vs 2002-2008)	1.188 (0.918-1.536)		.19		1.130 (0.879-1.453)			.34		
	Therapeutic hospital level (secondary vs tertiary)	1.033 (0.848-1.258)		.75		1.035 (0.851-1.259)			.73		
	Latency (≤12 months vs >12 months)	1.369 (1.103-1.699)		.004		1.339 (1.081-1.659)			.007		
	Number of PCs (with 3 PCs vs with 2 PCs)	1.041 (0.565-1.917)		.90		1.024 (0.556-1.885)			.94		

^a^Note: In the Cox regression for the first primary carcinoma site, 644 samples were missing values for the TNM stage variable (1560-916), so only 916 samples were included. For the second primary cancer, 716 samples (1560-844) were missing data; thus, 844 samples were analyzed.

^b^TNM: tumor, node, and metastases stage.

^c^PC: primary carcinoma.

## Discussion

### Principal Findings

This study provides the first comprehensive population-based mapping of MPC patterns in Shanghai, China, analyzing their occurrence, combinations, and survival using real-world registry data. By extending follow-up through 2017 and retrospectively including cases dating back to 1988, we obtained long-term observation that offers valuable insight into these relatively rare events. Moreover, the observed patterns and risk factors reflect fundamental biological and demographic influences, supporting their continued clinical relevance beyond the study period.

Our analysis of real-world data revealed that colorectal, stomach, and reproductive cancers (breast cancers in women and prostate cancers in men) were the most frequently diagnosed first PC. A previous study, which compared global cancer statistics data from China to other countries, found that among patients of both sexes, China had the largest proportion of lung, colorectal, and female breast cancer (38.9% of total cases; United Kingdom: 34.8%, United States: 29%), and also had a greater proportion of infection-attributable cancers (17.8% of liver and stomach cancers) [24]. Similarly, the results of our study indicate that the prevalence of digestive tract cancers, such as colorectal and stomach cancers, were higher [25]. Moreover, Feng et al [25] study on countrywide cancer prevalence found that 36.4% of Chinese cancer-related deaths were due to digestive tract cancers (stomach, liver, and esophageal cancer), compared to ≤5% of the total cancer-related deaths in the United States or the United Kingdom [25]. We concluded that primary lung cancer ranked first as the second PC site, but was the fourth most frequently observed PC in the first site, which does not agree with the findings of Feng et al [25] or with other previous studies, in which lung cancer has always ranked first. One reason for the difference in incidence rates between this study and prior studies may be the inadequate number of data sources and research on varying MPCs; thus, further study remains necessary. Additionally, sex-specific differences in MPC distribution were also observed. When organized by sex, the order of the top 10 PCs existed as the first and second PCs between male and female patients were distinct. Men had higher rates of infection-attributable cancers (stomach and liver), but women had higher rates of thyroid cancer. These patterns reflect the underlying differences in cancer etiology between sexes and may inform site-specific surveillance strategies for survivors with cancer.

When analyzing the PC combinations, we found that PCs including colorectal and lung, colorectal and stomach, colorectal and prostate, and breast and thyroid, breast and colorectal, stomach and colorectal, and stomach and lung were the most frequently observed. Moreover, it was common to observe comorbidities of the same system; for instance, there was a higher probability of cooccurrences of multiple digestive tract cancers (stomach and liver) [26]. Our results revealed patterns among the MPC that cooccurred; for instance, breast cancer appeared frequently with thyroid cancer. However, it is important to note that these observed patterns represent clinical cooccurrences whose frequency may be influenced by the high background incidence or survival rate of the involved cancers. Therefore, future studies incorporating standardized incidence ratios are needed to determine whether these observed combinations represent an elevated risk beyond chance. According to the study by Buikhuisen et al [10], concomitant diagnoses in the first year mainly comprised lung and renal cancer. Metachronous malignancies beyond the first year were most frequently observed for patients with breast, colorectal, prostate, and lung cancer [10]. Furthermore, a study of patients hospitalized at the Department of Oncology from 2003 to 2009 found that the incidence of MPC was 2.4%, and of the 103 patients with MPC, 97 had two, and 6 had three primary tumors. Moreover, the most frequently observed tumor combinations in men were prostate cancer-digestive system malignancies (especially colorectal cancer) and vice versa, as well as hematological malignant tumor-digestive system malignancies. In women, the most frequently observed multiple tumor occurrences were breast cancer, cancer of the contralateral breast, and hematological malignant tumors (especially non-Hodgkin lymphoma)-breast cancer. The observed associations between certain cancers are likely multifactorial in origin. Potential contributing mechanisms include shared hormonal pathways, common genetic susceptibilities, and environmental exposures. Additionally, the potential long-term effects of treatments received for the first PC should also be considered as a possible risk factor for subsequent malignancies in particular organs. Therefore, clinicians should be aware of these frequently observed combinations. Future analytical studies using standardized incidence ratios are warranted to confirm whether these pairs represent an excess risk beyond that expected by chance. Moreover, in this study, the 1-, 2-, and 3-year survival rates of patients with first or second PCs were higher, but the 4-year and 5-year survival rates were lower, which underscores the critical importance of sustained, long-term surveillance beyond the initial treatment period. Moreover, the survival rates of patients with breast and thyroid MPC were relatively high, which is consistent with the findings of previous studies [27]. Building on the patterns of metachronous cancer identified, our findings advocate for a paradigm shift in survivorship care, from a model focused solely on the primary tumor to a comprehensive, lifelong strategy. This necessitates a multidisciplinary approach that moves beyond the initial organ site to implement coordinated surveillance for the most likely subsequent cancers, based on the primary site, shared risk factors, and treatment history, thereby enabling earlier detection and improving long-term prognosis for survivors. For instance, given the high frequency of lung cancer as a second primary, future research should evaluate the potential role of enhanced surveillance strategies, such as low-dose chest computed tomography, in high-risk survivor populations.

Our results showed that, among the first PCs, skin and lung cancers had the lowest survival rates, which is consistent with the findings of other studies. For example, in Barton et al [28] systematic review, they found that the survival rates of nonmelanoma skin cancer, across both squamous cell carcinoma and basal cell carcinoma, tended to be poorer when it existed as a second PC. However, further study is required to more precisely characterize these associations and elucidate the underlying mechanisms. Patients with lung cancer as the first primary cancer also had relatively low survival rates, possibly because lung cancer is often not detected until it has spread; this finding is consistent with previous countrywide studies [29]*.* However, it should be noted that these survival estimates may be influenced by the retrospective design of our study. The Shanghai Cancer Registry achieved full population coverage in 2002, and patients with a first PC diagnosed before 2002 were required to survive until at least 2002 to be included. This introduced immortal time bias, which may lead to overestimation of survival for first primary cancers, especially for those diagnosed in earlier periods. Therefore, the absolute survival rates for first PCs should be interpreted with this limitation in mind. And future studies should use prospective designs or more uniformly enrolled cohorts to provide more precise survival estimates.

We observed low incidences of pancreatic and liver cancers as second primary cancers (ninth and eighth rankings, respectively), and the lowest survival rates among the most commonly occurring cancers. Pancreatic cancer often presents at an advanced stage, which contributes to poor 5-year survival rates of approximately 2%-9%, ranking firmly last among all cancer sites in terms of prognostic outcomes for patients [30]. Regarding liver cancer mortality in China, the mortality rate (422.1/1000) was lower than that of lung (610.2/1000) and stomach (498/1000) cancer, but higher than that of colorectal cancer (191/1000). Previous studies have found that liver cancer mortality is especially high in parts of Eastern China, such as Shanghai [22].

Notably, our study found that when the latency between the first and second PC diagnosis was ≤12 months, patients had a higher risk of mortality than those with an interval of >12 months. This finding indicates that the faster the new primary cancer occurs, the higher the mortality risk. Several mechanisms may underlie this association. First, a short latency period may reflect aggressive tumor biology or shared genetic susceptibility predisposing to the rapid development of a second PC. Second, treatment-related effects, especially the radiotherapy and chemotherapy administered for the first PC, may contribute to the development of second PC, with shorter latency potentially indicating greater genotoxic susceptibility or higher cumulative exposure. Third, surveillance bias and reverse causation may contribute, as patients with more frequent follow-up or more aggressive first PC are more likely to have a second PC detected earlier or incidentally. However, this seems to differ for nonprimary cancers, as patients must receive early diagnoses followed by chemotherapy and radiotherapy, which can help delay the occurrence of secondary cancer and mortality [31]. Further clinical analysis is needed to determine if there are differences in trends between different types of cancers.

This finding was different from those of other studies that researched nonprimary cancers, indicating that more attention should be given to the distribution of cancers by sex for the prevention of subsequent primary cancers. Moreover, female patients had higher mortality rates than male patients in the second PCs, but there was no significant difference between the first PCs. This finding appears to be different from previous studies on general Chinese cancer statistics, which have indicated that male patients have higher overall mortality rates [25]. In this study, patients >65 years old had higher mortality regardless of whether they existed in the first or second PCs than the younger. A prior study concluded that increased age correlated with higher rates of cancer diagnosis. Moreover, in recent years, the incidence of cancer in people >65 years old has been 10 times higher than that of people aged <65 years in the United States [32]. Furthermore, Ershler et al [33] found that cancer mortality rates were higher for older adults, with approximately 70% of all cancer-related deaths occurring in people aged >65 years. Consistent with other studies, our results showed that patients with stage III + IV cancer in both first and second sites had higher mortality. This is mainly due to the inability of these late-stage-diagnosed patients to receive early detection and treatment [34]. Also, it was found that patients diagnosed between 2009 and 2015 had a higher mortality. Correspondingly, there were more patients diagnosed between 2009 and 2015 in the III+IV stage, and more patients diagnosed between 2002 and 2008 in the I+II stage. However, due to the limited number of samples with multiple primary tumors, only 23 patients had third primary tumors. Therefore, we calculated the time based on the first diagnosis of each primary tumor. In addition, we found no significant differences between patients with 2 PCs and 3 PCs when comparing both cancer-specific and all-cause mortality. This finding is unique compared to previous non–primary cancer studies by Sarfati et al [31], which concluded that more cancer comorbidities led to higher mortality. However, further research is still needed to explore the effects of various kinds of cancer.

There are several limitations in this study. First, our sample was based only on data from the Shanghai Cancer Registry System, with a relatively small size [35-37], and stringent data governance policies after 2016 limited access to more recent cases. Therefore, future studies should include larger, updated samples as data sharing frameworks evolve. Second, the current registry data lack detailed information on lifestyle factors and genetic predisposition, which limits comprehensive risk factor analysis. Future studies integrating such data would help determine whether the observed cancer combinations share common hereditary or environmental causes. Third, a substantial amount of data, particularly on TNM staging, was missing from the registry, primarily due to delayed pathology reporting relative to cancer diagnosis. This led to the exclusion of cases from the Cox regression analyses, potentially introducing selection bias and reducing the statistical power of these models. Additionally, due to the insufficiency of the Cancer Registration System, patients with a first cancer before 2002 had to survive until at least 2002 to be included in the dataset, which may cause an overestimation of survival for first primary cancers and an overrepresentation of less aggressive cancer types in our observed MPC patterns. Fourth, our survival analyses employed standard Kaplan-Meier and Cox proportional hazards models. These methods may overestimate cancer-specific mortality in the presence of competing risks. Future studies with more granular cause-of-death data are encouraged to apply competing risk models to obtain more precise estimates. Additionally, in line with the descriptive aim of profiling cancer combinations, our models for the second primary cancer did not adjust for the site of the first PC, which did not isolate the independent effect of the second cancer itself. Future etiological studies may consider such adjustments.

### Conclusions

In this study, we provide comprehensive information synthesis from large, long-term datasets to improve the understanding of the prevalence of common MPC patterns and factors affecting survival status between various PC sites. Our results highlight specific cancer combinations with low survival rates, as well as risk factors, including a short interdiagnosis interval (≤12 months), advanced age (>65 years), and late tumor stage (III+IV). These results underscore the need for comprehensive prevention and control strategies toward the highly occurring MPC combinations in China and other countries, and also highlight the importance of clinical awareness directed toward the identified high-risk populations to enable earlier detection and intervention at an early stage of diagnosis.

## Appendix

Multimedia Appendix 1 RECORD checklist.

Multimedia Appendix 2 The patterns of all cancers.

Multimedia Appendix 3 The complete combination patterns of all cancers.

Multimedia Appendix 4 Kaplan-Meier curves of survival status and risk factors for first and second primary carcinoma (PC) patients.

## Abbreviations

CRC: colorectal cancer
HR: hazard ratio
*ICD-10*: *International Statistical Classification of Diseases and Related Health Problems 10th Revision*
MPC: multiple primary carcinoma
PC: primary carcinoma
RECORD: Reporting of studies Conducted using Observational Routinely collected health Data
SEER: Surveillance, Epidemiology, and End Results
STROBE: Strengthening the Reporting of Observational Studies in Epidemiology
TNM: tumor, node, and metastases stage
TRIPOD: Transparent Reporting of a multivariable Prediction model for individual Prognosis or Diagnosis

## Data Availability

The datasets analyzed during this study are available from the corresponding author on reasonable request and with permission of the related department.
