# Supplementary material for: Real-World Mapping of Multiple Primary Carcinoma Combinations and Survival Outcomes in Shanghai, China: Retrospective Registry-Based Study
Source: JMIR Public Health Surveill. 2026 Jul 6;12:e82355. doi: 10.2196/82355 (PMC13386119; doi:10.2196/82355)
Supplement: Multimedia Appendix 4 [file publichealth_v12i1e82355_app4.pdf]

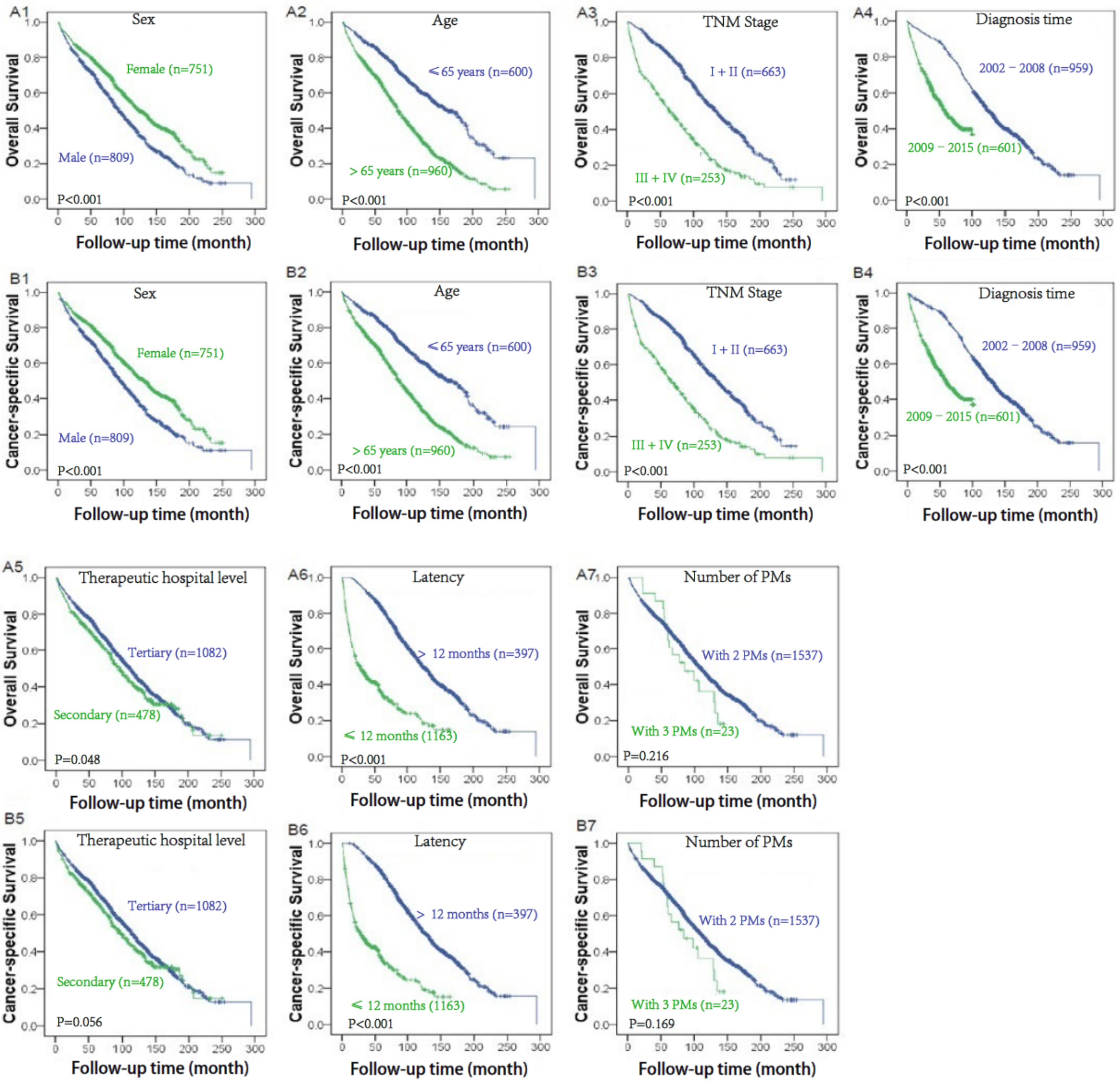

Supplement Figure 1a. Kaplan–Meier curves of the survival status and risk factors for first primary malignancy (PC) patients

Note: A indicates the overall survival for first PC patients, B indicates the cancer-specific survival for first PC patients

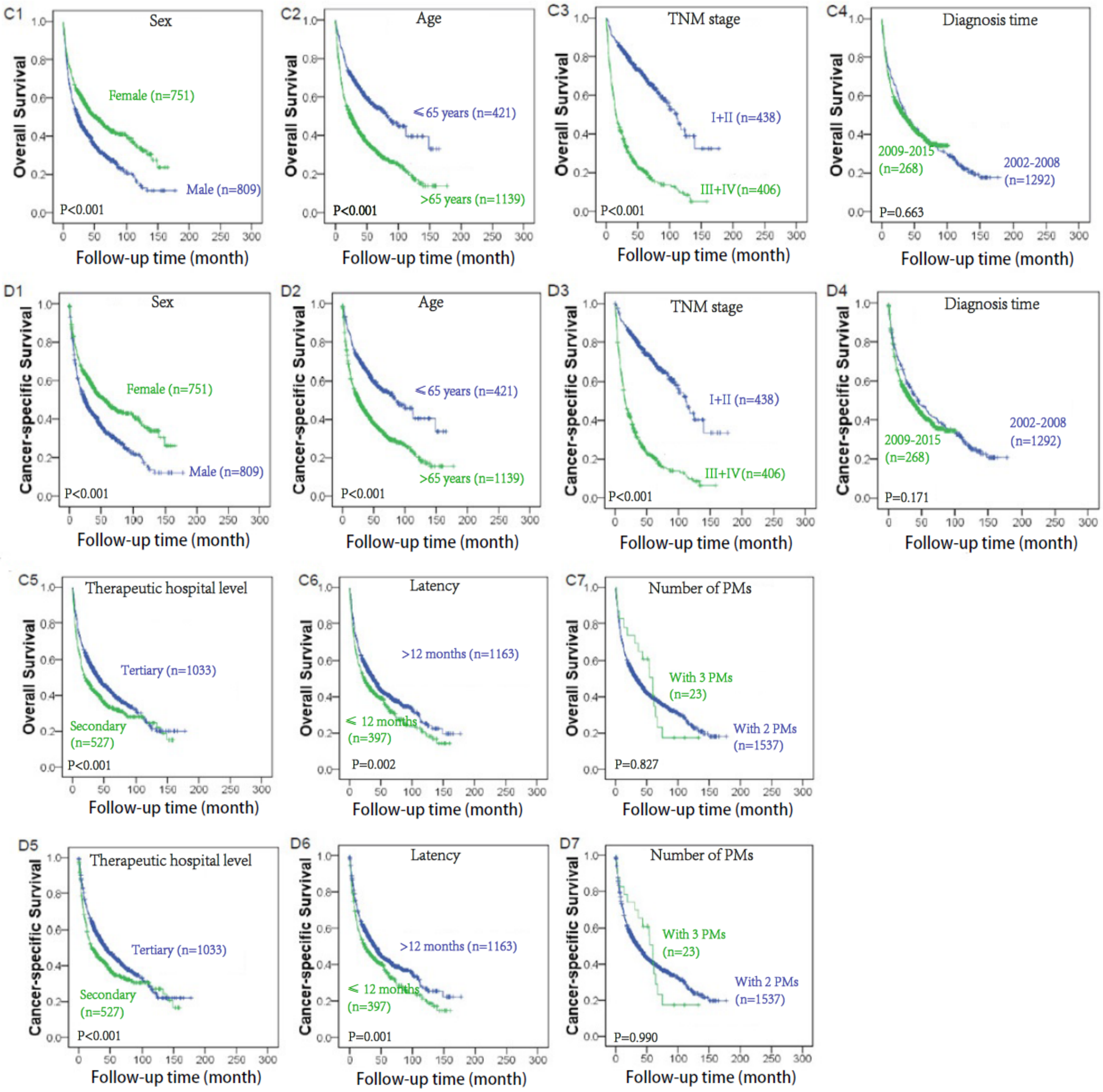

Supplement Figure 1b. Kaplan–Meier curves of the survival status and risk factors for second primary malignancy (PC) patients

Note: C indicates the overall survival for second PC patients, D indicates the cancer-specific survival for second PC patients
